# First-pass perfusion CMR with reduced dark-rim artifact and instantaneous image reconstruction using optimized cartesian sampling and apodization

**DOI:** 10.1186/1532-429X-17-S1-P1

**Published:** 2015-02-03

**Authors:** Zhengwei Zhou, Xiaoming Bi, Hsin-Jung Yang, Rohan Dharmakumar, Reza Arsanjani, Noel Bairey C Merz, Daniel S Berman, Debiao Li, Behzad Sharif

**Affiliations:** 1 Biomedical Imaging Research Institute, Cedars-Sinai Medical Center, Los Angeles, CA, USA; 2 Department of Bioengineering, University of California, Los Angeles, Los Angeles, CA, USA; 3 Cedars-Sinai Heart Institute, Los Angeles, CA, USA; 4 MR R&D, Siemens Healthcare, Los Angeles, CA, USA

## Background

Subendocardial dark-rim artifact (DRA) continues to be a major issue that limits the diagnostic performance of first pass perfusion (FPP) CMR. Non-Cartesian approaches such as radial or spiral acquisition have been proposed to minimize DRAs. Among these approaches, those that can operate without the need for breath-holding typically require a time-consuming offline image reconstruction procedure, which limits their clinical accessibility. We propose a free-breathing DRA-reduced FPP scheme with instant image reconstruction on the scanner.

## Methods

Healthy subjects (n=10) were studied on a 3T Siemens scanner using the standard 12-channel coil. Two free-breathing FPP scans (3 slice coverage) were performed at rest, first using the proposed method followed by a conventional method (15 minute gap).

Apodization of k-space data is known to reduce Gibbs ringing effects, a major underlying cause of the DRA. It also enhances signal-to-noise (SNR) but comes at the cost of lower resolution. In the proposed method, a high parallel imaging factor (4 fold) with Cartesian sampling is used to acquire a high-resolution FPP scan (in-plane: 1.7 x 1.7 mm^2^). Following parallel imaging reconstruction, an optimized apodizer is applied, which both reduces Gibbs ringing and improves SNR. The apodization level was adjusted so that the apodized images had the same resolution as the conventional FPP scan (in-plane: 2.7 x 2.2 mm^2^).

Apodization was implemented as an additional post-filtering step and it was integrated to the inline image reconstruction routine of the scanner. Images were scored by 2 expert readers blinded to the protocol (consensus artifact scoring; scale: 0 = No DRA, 4 = Severe DRA). In addition, dogs with myocardial infarction (n=2) and a control dog were studied using a similar setting.

## Results

**Figure 1:**
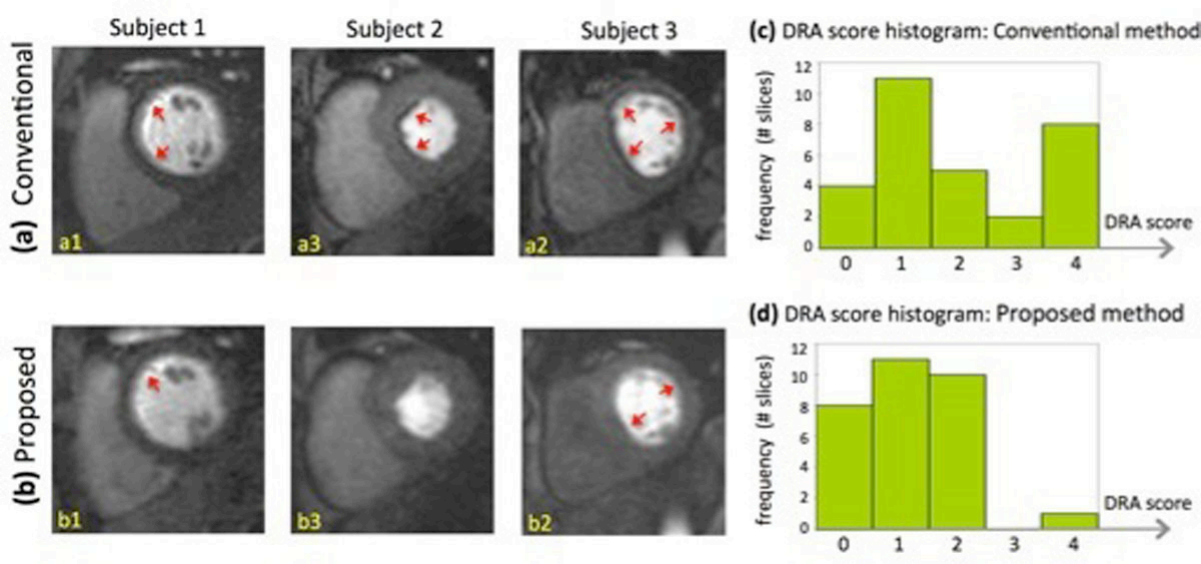
Representative rest FPP images of three healthy subjects. Images in the top panel **(a1-a3)** correspond to the latest vendor-provided conventional FPP method and the bottom row **(b1-b3)** correspond to the proposed perfusion CMR technique. All images are in the same myocardial enhancement phase. As can be seen, the number of segments affected by DRAs and their severity is reduced in the proposed method compared to the conventional method. **(c,d)**: Histograms of DRA scores for the proposed and conventional methods. DRA is significantly reduced in FPP images acquired with proposed method (1.2 versus 2.0; p<0.01). Importantly, the number of slices with severe DRA (artifact scores = 3 or 4) is significantly lower for the proposed method (by 10 fold) as can be observed by comparing (c) and (d).

Fig. 1(a,b) shows representative images and the histograms of DRA scores are presented in Fig. 1(c,d). The mean DRA score for the proposed method is lower than the conventional method (p<0.01). More importantly, the number of slices with severe DRA (scores 3 or 4) is significantly lower for the proposed method. Image quality scores were also assigned by the readers (0-4 scale; 0: poor; 4: excellent). The difference of image quality between the two methods was not significant (3.5 for the proposed method and 3.9 for conventional; p=0.2).

**Figure 2:**
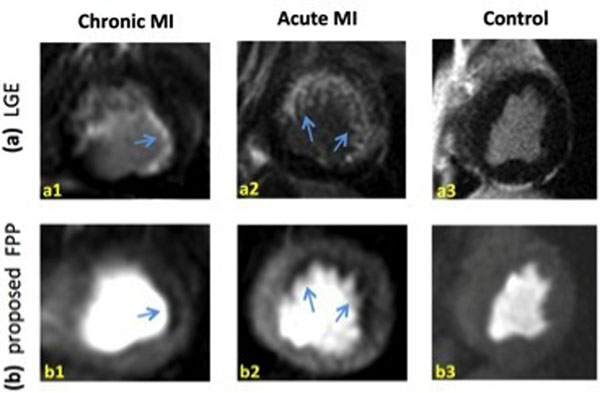
Representative FPP images and corresponding late gadolinium enhancement (LGE) images in canine models of myocardial infarction (MI). **(a1,b1):** images correspond to a chronic MI dog study that shows small subendocardial LGE (a) and FPP defect in the lateral wall. The defect is clearly seen demonstrating the ability of the proposed method to resolve small defects (following apodization). **(a2,b2)** Similar result with a larger defect region in an acute MI dog. **(a3,b3):** images for a control animal study. All images correspond to the peak myocardial enhancement phase. Among the 3 studies, minimal DRA is seen in only (b2) and (b1,b3) are free of DRA. The results demonstrate that reduction of subendocardial DRAs using apodization (applied to a high-resolution scan) does not lead to elimination/masking of subendocardial defects in the reconstructed apodized images.

Fig. 2 provides results from the canine studies, demonstrating that FPP images acquired with proposed method can detect small subendocardial defects corresponding to infarct territories (LGE images).

## Conclusions

We developed an optimized Cartesian sampling and apodization scheme for free-breathing perfusion CMR. Compared to the conventional FPP imaging method, the severity and prevalence of subendocardial DRAs were significantly reduced. A key feature of the proposed method is that it uses the widely-available parallel imaging reconstruction software to achieve near-instant reconstruction on the scanner, potentially enhancing its accessibility in clinical studies.

## Funding

Grant sponsors: NIH National Heart, Lung and Blood Institute grant nos. K99 HL124323-01, R01 HL038698-18, R01 HL091989-05, R01 HL090957-01; and the Barbra Streisand Women's Cardiovascular Research & Education Program, CSMC.

